# CREM perspective on home office—a consideration of the workplace and its mechanisms of action

**DOI:** 10.1365/s41056-022-00060-4

**Published:** 2022-06-27

**Authors:** Kyra Voll, Felix Gauger, Andreas Pfnür

**Affiliations:** Technical University of Darmstadt, Hochschulstraße 1, 64289 Darmstadt, Germany

**Keywords:** Home office, CREM, Productivity, Workplace, PLS-SEM, Heimarbeit, CREM, Produktivität, Arbeitsraum, PLS-SEM

## Abstract

The effect between the workplace and work success is a black box whose mechanisms have so far received little theoretical substantiation. In the explanation of the importance of corporate real estate and its management for the success of companies, the influence of real estate on the work productivity of employees through the physical workplace is shown. However, the overall picture has not yet been fully elaborated and the fragmentary knowledge is only partially suitable for attributing organizational outcomes to the characteristics of the physical working environment. Without sufficient empirical data and a solid theoretical foundation for physical working environment studies, it is not possible to draw conclusions with sufficient certainty about the impact of working environments on organizational outcomes.

The fact that millions of people worldwide are working from home for the first time during the COVID-19 pandemic provides an unprecedented opportunity to explore the impact of the home office environment on business success.

This study aims to contribute to filling this research gap by further investigating the impact of the physical working environment at home on productivity by building on the Environmental Demands–Resources model. Therefore, the research goal is to determine which of the four included demands and resources (isolation, family–work interference, equipment/facilities, and building) have an impact on employee burnout and satisfaction, and how this impact affects employee productivity. Partial least squares structural equation modeling is used to analyze a German survey sample (*n* = 429).

The results suggest that the four included workplace characteristics have significant influence, with equipment/facilities and building increasing satisfaction and isolation and family–work interference increasing burnout. Equipment/facilities is identified as the most important factor affecting productivity in the home office.

Through this study, a contribution is made to establish a more inclusive and integrative framework for physical working environment research. In addition, the results show that workspace characteristics have an impact on productivity. Far beyond the pandemic, the impact of changes in workspace design on employee perceptions and organizational performance will be important to corporate real estate management practice.

## Abstract (de)

Die Wirkung zwischen Arbeitsplatz und Arbeitserfolg ist eine Blackbox, deren Mechanismen bisher wenig theoretisch untermauert wurden. Bei der Erläuterung der Bedeutung von Unternehmensimmobilien und deren Management für den Erfolg von Unternehmen wird der Einfluss von Immobilien auf die Arbeitsproduktivität von Mitarbeitern durch den physischen Arbeitsplatz aufgezeigt. Allerdings ist das Gesamtbild noch nicht vollständig ausgearbeitet, und das fragmentarische Wissen ist nur bedingt geeignet, organisatorische Ergebnisse auf die Merkmale der physischen Arbeitsumgebung zurückzuführen. Ohne ausreichende empirische Daten und eine solide theoretische Grundlage für Studien zur physischen Arbeitsumgebung ist es nicht möglich, mit ausreichender Sicherheit Schlussfolgerungen über die Auswirkungen der Arbeitsumgebung auf organisatorische Ergebnisse zu ziehen. Die Tatsache, dass Millionen von Menschen weltweit während der COVID-19 Pandemie zum ersten Mal von zu Hause aus arbeiten, bietet eine noch nie dagewesene Gelegenheit zur Erforschung der Auswirkungen des Home Office auf den Unternehmenserfolg.

Der Literatur über die physische Arbeitsumgebung fehlt eine theoretische Fundierung, um organisatorische Ergebnisse auf die Merkmale der physischen Arbeitsumgebung zurückzuführen. Die vorliegende Studie soll dazu beitragen, diese Forschungslücke zu schließen, indem sie aufbauend auf dem Environmental Demands-Resources-Modell die Auswirkungen der physischen Arbeitsumgebung zu Hause auf die Produktivität untersucht. Ziel der Studie ist es einen Beitrag dazu zu leisten die Wirkungsmechanismen des Arbeitsorts auf den Arbeitserfolg empirisch zu erklären. Dazu werden vier Anforderungen und Ressourcen des Heimarbeitsplatzes auf ihren Einfluss auf Burnout und Zufriedenheit der Mitarbeiter und deren Einfluss auf die Produktivität der Mitarbeiter überprüft. Zur Analyse einer deutschen Umfragestichprobe (*n* = 429) wird die Strukturgleichungsmodellierung verwendet.

Die Ergebnisse belegen, dass die vier einbezogenen Arbeitsplatzmerkmale einen signifikanten Einfluss haben, wobei die Ausstattung/Einrichtungen und das Gebäude die Zufriedenheit erhöhen und die Isolation und die Überlagerung von Familie und Arbeit Burnout erhöhen. Ausstattung/Einrichtung wird als wichtigster Faktor identifiziert, der die Produktivität im Home Office beeinflusst.

Mit dieser Studie wird ein Beitrag zur Erklärung der theoretischen Wirkungsmechanismen zwischen Arbeitsort und Arbeitserfolg geleistet. Die Ergebnisse zeigen, dass die vier untersuchten Merkmale des Arbeitsplatzes zuhause einen Einfluss auf die Produktivität der Mitarbeiter haben. Somit wird deutlich, dass physische und sozialpsychologische Faktoren des Heimarbeitsplatzes den Arbeitserfolg maßgeblich mitbestimmen.

## Introduction

In times of real estate transformation and the trend toward work-from-anywhere, it is highly relevant for decision-makers in corporate real estate strategy to understand the mechanisms between the workplace and work performance. Therefore, factors that influence employees’ productivity when they work from home have to be identified and empirically tested to adapt corporate real estate management (CREM) strategies effectively. The COVID-19 pandemic has forced companies abruptly to change the way work is executed and enable their employees to work from home (Kramer and Kramer 2020). Organizations’ CREM departments must address the new situation with a large share of people working in the new home office work environment instead of the office. Employees must utilize the resources and cope with the demands that exist at home in contrast to their usual work environment in the office. In addition, while working from home, the boundaries between private life and everyday work life become blurred, increasing employee exhaustion and the risk of burnout (Gigauri 2020). Basic human requirements for the working environment must be met to ensure productive work and commitment to the company (BMFSFJ 2017). From the employee’s perspective, an environment that is optimally suited to their work is beneficial to complete tasks effectively (Armitage and Nassor 2021). Well beyond the pandemic, the impact of new and flexible workplaces, such as the home office on employee perceptions and organizational performance, will continue to be relevant for CREM business practices (Alipour et al. 2020).

In recent years, research has been conducted to prove the relevance of the classic office environment as an operational resource for organizational outcomes like employee satisfaction and performance (Krupper 2013). The New Ways of Working (NWW) approach is introduced in many organizations worldwide, dedicated to flexible work design and enabled by information and communication technologies (ICTs) (Blok et al. 2011; Nijp et al. 2016). The definitional framework of NWW includes telework and home-based work (Blok et al. 2011). While positive effects for organizations have already been demonstrated for telework (Harker Martin and MacDonnell 2012; Bloom et al. 2015), research on home office is just gaining momentum during the COVID-19 pandemic (Fadinger and Schymik 2020). Even if it can be assumed that the home office as a new working environment, similar to the classic office, working environment influences organizational outcomes through the employee satisfaction, scientific proof is lacking.

Millions of people worldwide are working from home for the first time, providing an unprecedented opportunity for research on the resulting impact of the workplace at home on work success (Contreras et al. 2020). Such research is of great interest and importance to effectively understand this new way of working and manage corporate real estate more effectively and efficiently (Donthu and Gustafsson 2020). To measure the influences of NWW, several different model approaches exist. Individual studies are typically segmented in different facets by discipline and not linked to a conceptual framework (Clippard 2020; Pfnür et al. 2021). However, the overall picture has not yet been fully elaborated and the fragmentary knowledge is only partially suitable for attributing organizational outcomes to the characteristics of the physical working environment (Pfnür et al. 2021). Without sufficient empirical data and a solid theoretical foundation for physical working environment studies, it is not possible to draw conclusions with sufficient certainty about the impact of NWW on organizational outcomes (Blok et al. 2012).

This paper aims to contribute to filling this research gap by further investigating the mechanisms of action of the physical working environment home office on organizational outcomes during COVID-19 by building on the theoretical foundation of the Environmental Demands–Resources (ED–R) model (Roskams et al. 2021) as an application of the Job Demands–Resources (JD–R) model (Bakker and Demerouti 2007). Therefore, the research goal is to determine which work characteristics in the home office have an impact on employee burnout and satisfaction, and how they affect employee productivity. Partial least squares structural equation modeling (PLS-SEM) is used to analyze the relationships within data collected from a German Work-from-Home survey sample (Pfnür et al. 2021).

The results add to the literature of CREM and physical environmental research by replicating the ED–R model to offer new approaches of model extension and establish a more inclusive and integrative framework for physical working environments. CREM can use the results to gain knowledge about home offices as substitutes and complements for the traditional office, and to understand the mechanisms of action of various workplace characteristics on organizational outcomes that will subsequently enable better management of real estate as an operational resource.

## Theoretical background

### The changing world of work forces NWW with more flexibility

In addition to the COVID-19 pandemic, the world of work is constantly exposed to new challenges due to the social, economic, and technological developments of the last 20 years (Cascio 2010; Gauger and Pfnür 2019). The demographic and social development changes in the work force in Germany have led to the need for organizational changes.

Pfnür (2019) and Pfnür and Wagner (2020) show in their empirical studies on real estate transformation that German corporations will adapt 60% of their operational real estate to changed conditions of use within 10 years through project development and market transactions as a result of the megatrends such as demographic change, digitalization, globalization, urbanization, growing environmental sensitivity, and increasing government intervention. Work-from-home settings offer CREM additional opportunities to respond quickly and flexibly to changing space requirements. In addition, the physical workspace is increasingly augmented by a digital space (Kellner et al. 2020). However, it has not yet been clarified what impact such innovative workplace concepts have on work success.

In dealing with the challenges in the world of work framework, significant adjustments are important in the organization and office space that impact on employees (Howard 1995; Holman and Wood 2003; Niessen et al. 2010). Flexibility in working life is increasingly demanded in the context of unpredictable social or technological changes as Murphy and Jackson (1999) state in their article on the challenges of the 21st century for organizations and employees. Unlike traditional systems, which are often less able to respond to challenges, flexibility enables rapid responsiveness. In a global context, the integration of flexible forms of work can be done by the establishment of NWW. NWW is an approach introduced in many organizations worldwide, enabled by ICT and characterized by factors that play a role in achieving productivity and business success in the wake of new challenges (Blok et al. 2011; Nijp et al. 2016). Those new types of work organizations (Nijp et al. 2016) with new workplace design strategies have effects on CREM. In literature, the definitional framework of NWW includes the term “working from home” (Blok et al. 2011) as it represents an implementation option of location-flexible ways of working. In order to meet the emerging demands through flexible forms of work, comprehensive design from an occupational science perspective is inevitable and conservative work structures must be disassembled (Ahlers et al. 2018). Workplace flexibility itself has many facets (Hill et al. 2008). In addition to working hours, for example, flexible work arrangements are often characterized by spatio-temporal flexibility (Allen et al. 2013; Schulze et al. 2015). Spatio-flexible approaches enable employees to choose the place where they perform their tasks during working hours (Chen and Fulmer 2017). While flexibility has been previously viewed primarily as an accommodation to employees, this impression has changed. Companies, including their CREM, have recognized that flexibility offers potential benefits for them, for example, in terms of cost savings and business attractiveness (Pitt-Catsouphes and Matz-Costa 2008). Moreover, flexible ways of working through ICTs and higher utilization rates of flexible uses lead to greater resource efficiency (Gauger and Pfnür 2019).

### Evolvement of home office as work environment and opportunity for CREM strategies

The workplace as a place of meaningful employment is subject to constant change. Its design should be adapted to the new forms of work that accompany the constant changes in the world of work triggered by changing needs and requirements of the place where work is performed (Coles 2011). The broad term “work environment” includes aspects of the physical environment at work, such as work equipment as well as psychological aspects, like work organization and satisfaction at work. The physical aspect of the place of work is usually the first to be considered. In relation to this, a valuable contribution that a work environment can make to a company’s success is to support people in their work in the best possible way through suitable locations (Armitage and Nassor 2021).

Before industrialization, living and working usually took place under one roof. This often-prevailing unity of living and working was broken up by the development of the agrarian state into an industrial state. Oldenburg (1999) posits that for a healthy existence, citizens must live in a balance of three areas, which he refers to as home life, the workplace, and inclusive social places. Referring to the individual home, he coins the term “first place.” Later in history, an increasing share of knowledge-intensive activities for a large part of the workforce spatially dissolved the connection between life and work. Since then, so-called “knowledge workers” spent their working time in offices, also referred to as “second places” (Oldenburg 1999). Since 1980, statements of workers and employees have been circulating that there is no longer any need for knowledge workers to work entirely at the workplace as soon as a computer is available to them on a mobile basis for any location (Johns and Gratton 2013). Technologization and emerging flexible work models have expanded the range of optional work locations. In connection with the emerged location-flexible possibilities to work, so-called “third places” like coworking spaces, also can be considered as workplaces. In recent years, the increasing flexibilization of the work location offers complementary alternatives to the office workplace. The former first place, the home, receives increasing attention as an office. Especially during the COVID-19 pandemic employees must adopt a way of working that is new to many (Contreras et al. 2020). Working from home requires employees to shift most of their activities from the physical space in the office to their home and a digital space in which they can act and communicate (Kellner et al. 2020).

With work no longer being done exclusively from the office, CREM strategies need to be adopted. On the one hand, companies are forced to cut costs, which has so far often been achieved through greater space efficiency; on the other hand, the focus can be shifted toward investments in the work environment for the workforce. Pfnür et al. (2019) provide with their framework the three mechanisms of CREM performance—operating, real estate, and financial performance—as a starting point in explaining the impact of corporate real estate resources and management on real estate-related company performance. In this framework, it becomes clear that user-related values of the operating performance of CREM, through value in enhancing employee satisfaction with the workplace and enhancing labor productivity, contribute to success at the workplace level. As reported in a literature review by De Croon et al. (2005), companies see an opportunity in the promotion of performance and increase of satisfaction of their employees by workspace design to achieve economic success. If the value of the space can be increased for the users, then additional costs may be outweighed by the improved organizational outcomes. This change in thinking is supported by knowledge about the effect of optimized workspaces for employees who experience increased satisfaction and productivity. The literature shows that specific design (Appel-Meulenbroek et al. 2020), comfortable spaces (Mariotti et al. 2021), and the configuration of spaces (Orel and Del Alonso Almeida 2019) are the main factors for optimizing employee satisfaction. Moreover, several studies already prove the relevance of the spatial work environment as an operational resource on work satisfaction and performance (Moleski and Lang 1982; Feige et al. 2013; Kim et al. 2016; Hoendervanger et al. 2018). With the real estate transformation and the trend toward work from anywhere, it is now important to empirically prove which sub-aspects of the physical workplace of the new working environment at home influence work success by promoting satisfaction and productivity.

## Hypotheses development

**Fig. 1:**
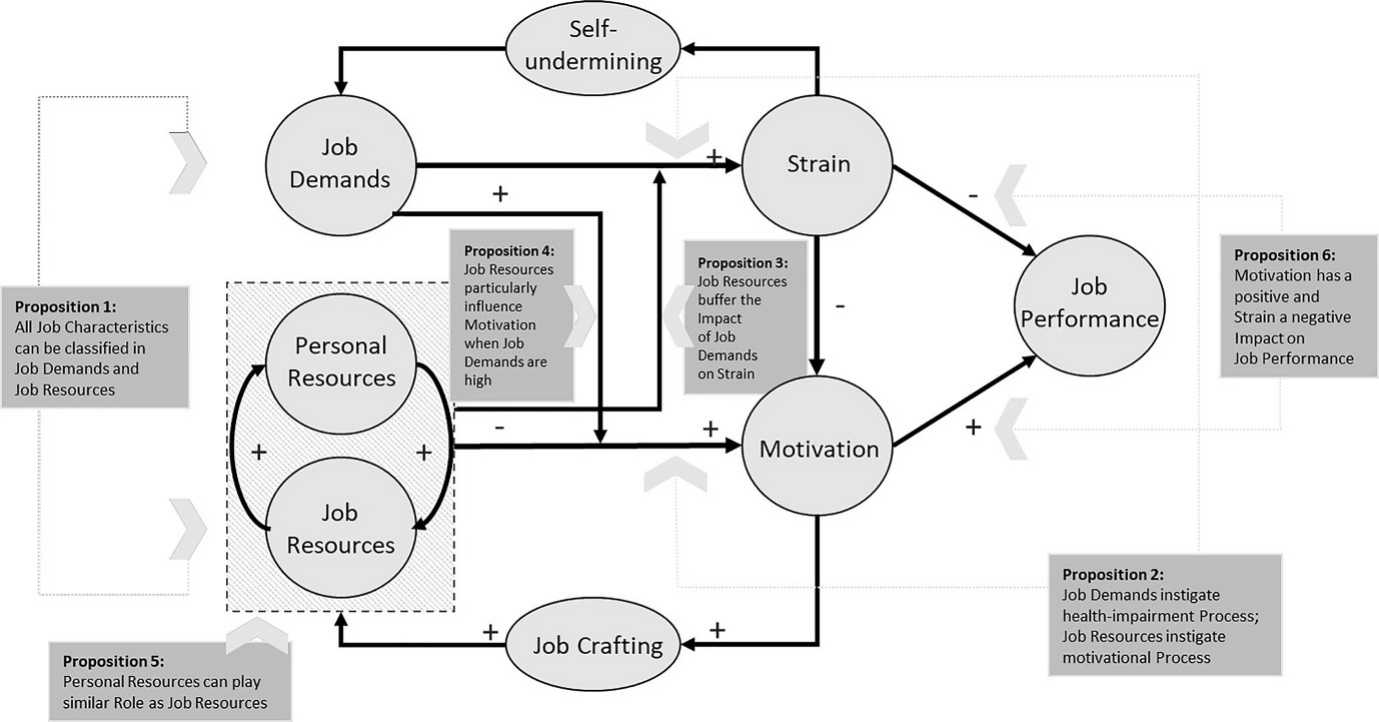
Job Demands–Resources Model. (Own Illustration 2021 following Bakker and Demerouti 2017)

A variety of research disciplines have examined environmental conditions, interactions, and success factors of office real estate on the workplace as well as of the physical work location on employee performance (e.g., Appel-Meulenbroek et al. 2013; Clippard 2020; Roskams et al. 2021). This research can serve as a basis for analyzing the home office workplace from a CREM perspective. The JD–R model is one of the most popular and influential models of work stress in organizational literature (Demerouti et al. 2001; Bakker and Demerouti 2017). The JD–R model describes the interaction of work-related resources (e.g., criteria of humane work design) and demands (e.g., environmental stressors), and states several propositions (see Fig. 1).

**Fig. 2:**
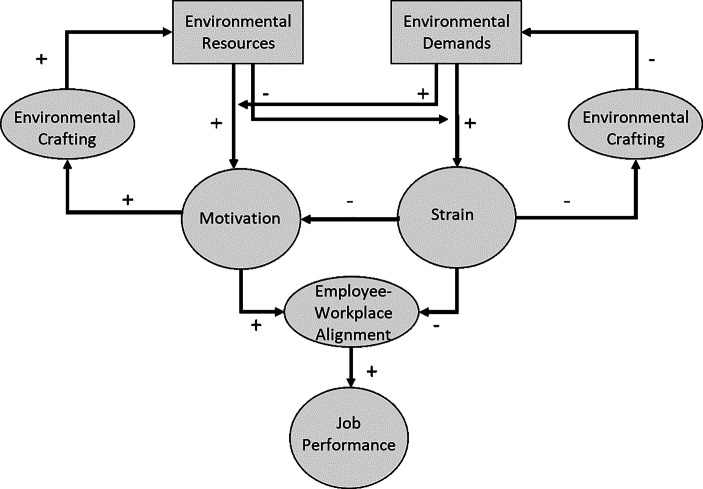
Environmental Demands–Resources Model. (Own Illustration 2021 following Roskams et al. 2021)

In addition to the general design and propositions of the JD–R model, the ED–R model, as applied to the workplace environment, is used (see Fig. 2). The overall idea of the ED–R model is presented by demands and resources determining the level of alignment between the employee and the workplace, which itself has a positive impact on human flourishing and increases job performance. Building on research measuring mental health in positive terms rather than by the absence of mental illness (Keyes 2002), human flourishing is defined as life within an optimal range of human functioning (Fredrickson and Losada 2005). The main difference between the JD–R and the ED–R models is the two broad categories into which the characteristics are divided. Environmental demands and resources are aspects of the workplace environment whose effects are like those of job demands and resources. Both categories in the process flow of the model subsequently influence job performance. The decision to base the research model of this paper on both theories is based on the effort to promote the theoretical approach with application to the workplace environment and on solid theoretical foundation for further studies on the physical work environment.

**Fig. 3:**
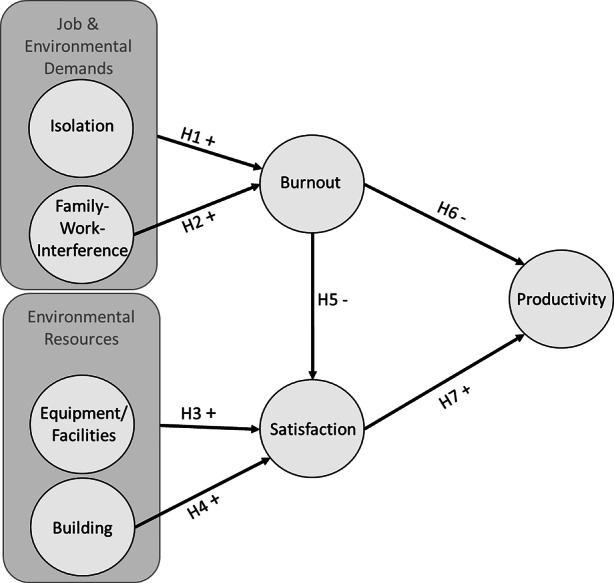
Research Model. (Own Illustration 2021)

This paper addresses the following research question: “Which of the four resources and demands of home office (isolation, family–work interference, equipment/facilities, and building) have an impact on employee burnout and satisfaction and how does this impact affect employee productivity?” All four workplace characteristics are consciously selected because they are within CREM’s scope of action and undergo a change in content or strength of relevance due to the pandemic condition (see Fig. 3).

Isolation can take on the quality of potential stressors as a burden of the working environment from the so-called “behavior setting” (Richter and Hacker 1998). An employee no longer leaves his home to perform work activities but does so from his private premises. Studies have identified social isolation as a hazard of telework (e.g., Baruch 2000). For single workers, a significantly reduced rate of physical contact in the COVID-19 pandemic due to working at home and the additional constraints of private and public life takes place (Huxhold and Tesch-Römer 2021). When employees work from home, they usually work alone and no longer have any physical social exchange with colleagues during their working hours. Because of their physical isolation in their home office, these employees experience loneliness and feelings of isolation (Wang et al. 2021). Bloom et al. (2015) identify this loneliness and a lack of social interaction while working from home as the most common reason why employees wish to return to the office. If home-based work takes place over a long period and with high intensity, then adverse factors, such as social isolation, occur more frequently (Allen et al. 2015). The pandemic lockdown phases in Germany in 2020 count as the first long-lasting, widespread spread establishment of home-based work, which means that the isolation is particularly strong (Huxhold and Tesch-Römer 2021; Wang et al. 2021). Isolation is found to be a variable that works in concert with stress created by work expectations in the form of role ambiguity, role overload, and role conflict, which have implications for job satisfaction and depression (Dussault and Thibodeau 1997; Izgar 2009). Demerouti et al. (2001) state that job demands are primarily related to the exhaustion component of burnout. This assumption is confirmed by results documenting a direct relationship between isolation and burnout (Bauer and Silver 2018). Based on the theoretical perspectives and empirical evidence, the first hypothesis is:

### H1

Isolation has a positive impact on burnout in home office.

Family conflicts, as potential stressors, can take on the quality of a strain on the work environment from the so-called “person system” (Richter and Hacker 1998). Working from home increases the risk of blurring the boundaries between work location and private life (Wang et al. 2021). In the COVID-19 pandemic, employees who do not live alone in a household but share it with a partner or family have different people around them during working hours than in the office. Instead of colleagues, there are partners and or children or even people in need of care, if necessary, physically in the place where the work activity is performed. As early as 1985, critics of home-based work who feared spatial fusion argued that the development of separate settings for home and work activities has led to balanced relationships in family and society that should not be broken up (Ahrentzen 1990). Family–work interference is a rather underexplored hindrance stressor (Turner et al. 2014). In home office, employees can experience all three family–work conflict types: time-based, strain-based, and behavior-based (Greenhaus and Beutell 1985). The time needed to fulfil work performance may be missing in the family area. The missing time is exacerbated by a possible multiplication of non-work-related tasks as the combination of work and tasks at home is concentrated in the private premises. In addition, the stress of work or family life can interfere with the performance of each role and it is possible that the two behavioral patterns desired in the different domains (e.g., concentrated work or frequent help with children’s homework) are incompatible (Greenhaus and Beutell 1985). Ultimately, the unsuccessful attempt to maintain functioning of both domains can lead to psychological distress and potential performance losses (Turner et al. 2014). During the COVID-19 pandemic, challenges for working parents, such as caring for children and assisting them with home schooling, arise when childcare options are eliminated. This is because the lack of childcare options further exacerbates the conflict between the role in the family and the role at work. A literature review by Eby et al. (2005), which examines the consequences of family interference with work, indicates that employees with high levels of family–work interference experience more stress. Frequent distractions and interruptions by cross-domain roles lead to greater experiences of exhaustion (Kreiner et al. 2009). When working from home, this can also be exacerbated by the feeling that one must be permanently available to the employer (Eurofound and the International Labour Office 2017). In addition, empirical evidence suggests a relationship between stressful events in personal life and job burnout (Hakanen and Bakker 2017). A meta-analysis by Alarcon (2011) confirms role conflict as a predictor of burnout. Hypothesis two, therefore, is as follows:

### H2

Family–work interference has a positive impact on burnout in home office.

The original conceptualization of the JD–R model classified an adverse work environment as a potential job demand (Demerouti et al. 2001). Nevertheless, before the establishment of the ED-R model, few studies addressed environmental factors and their impact as a demand or resource (Hakanen et al. 2005; Morrison and Macky 2017; Roskams and Haynes 2021). However, the design of office space contributes to the success of the company (Appel-Meulenbroek et al. 2013). Office building design focuses on the design and functionality of modern furniture and high-tech ICT. High-tech ICT supports a smooth flow of activities in times of digital work. NWW approaches are based on good access to information and knowledge (Eurofound and the International Labour Office 2017). Thus, while working from home, one of the most important prerequisites for efficient work is functioning technology (Messenger and Gschwind 2016). Bakker et al. (2003) includes problems with equipment as job demand. Van der Voordt (2004) recognizes a positive correlation between access to needed technology and satisfaction. A primary interest in home office lies in functional technical equipment; its presence is classified as a job resource, which stimulates a further development beyond the previous way of working (Messenger and Gschwind 2016). Moreover, a concept called “workplace performance” is increasingly emerging among researchers with the explicit goal of supporting job performance because a high-performing workplace should optimize employee productivity (Clements-Croome 2006). The aforementioned aspects and their impacts are reflected in the following hypothesis:

### H3

Equipment and facilities have a positive impact on satisfaction in home office.

In addition, ergonomic workstations and an attractive and stylish layout, for example, contribute significantly to a positive evaluation by users (Van der Voordt 2004). With respect to purely spatial factors, the greatest potential contributor toward physical discomfort and dissatisfaction is the ergonomic design quality. Thus, Roskams and Haynes (2021) group problems with ergonomic design as “environmental demand.” Research also demonstrates that some indoor environmental quality baseline factors in buildings have a negative impact on satisfaction when they are found to be inadequate (e.g., Sundstrom et al. 1980; Kim and De Dear 2012). These include, among others, the amount of space available, the adjustability of furniture, and architectural correlates, like privacy. Looking at the research results the other way around, it means that the private physical space perceived by workers as sufficient and adaptable and an ergonomic workstation in the home office positively influence satisfaction. Hence, it is posited as follows:

### H4

Home has a positive impact on satisfaction in home office.

The absence of burnout is considered as one of the most important predictors of job satisfaction (Lu and Gursoy 2016). The health impairment process is equated with burnout in this research model (e.g., Hakanen et al. 2006, 2008; Crawford et al. 2010). The motivational process is characterized as satisfaction. The underlying logic follows social exchange theory, which provides an explanation of the relationship between employee and organization (Rousseau 1995). An employee who feels supported by his organization reciprocates by showing desirable work attitudes such as higher satisfaction (Guimaraes and Dallow 1999). Depending on whether employees perceive working from home during the COVID-19 pandemic as beneficial or not, perception may subsequently change their mental state and attitude toward the organization. In terms of job-related outcomes, different meta-analyses and studies show evidence for the negative relationship between burnout, especially emotional exhaustion, and job performance (Wright and Bonett 1997; Taris 2006). Analyses of predictors and consequences of job burnout (e.g., Demerouti et al. 2001; Kim et al. 2009) show that it is correlated with a variety of negative responses to the job in various fields (e.g., Schaufeli and Enzmann 1998; Maslach and Jackson 1986; Alarcon 2011). Based on Conservation of Resources (COR) theory, burnout reduces an employee’s required resources, leading to dissatisfaction and a sense of ineffectiveness, and subsequently to turnover. The COR theory is not explicitly discussed in detail in this work and only serves as an underlying evidence base for the suitability of the JD–R model; therefore, it appears as relevant content in this paper. Vischer (2008) confirms that due to the increased need to expend energy to compensate for environmental conditions, such job demands, can lead to burnout. Burnout, in turn, can cause functional discomfort, such as interference with the successful completion of work-related activities in the concept of job stress. A number of studies find empirical evidence for a negative causal relationship of burnout to job satisfaction (Wolpin et al. 1991; Baruch-Feldman et al. 2002; Ybema et al. 2010). Two hypotheses are developed to test whether the same consequences of burnout occur for home-based work:

### H5

Burnout has a negative impact on satisfaction in home office.

### H6

Burnout has a negative impact on productivity in home office.

Research on telecommuters has shown that an increase in satisfaction supports higher productivity (Dubrin 1991). Moreover, in the “Happy-Productive Worker Paper” (Landy 1985), revisited by Zelenski et al. (2008), the author finds a positive relationship between job satisfaction and productivity. Based on the evidence provided the effects of satisfaction are tested for home office by the following hypothesis:

### H7

Satisfaction has a positive impact on productivity in home office.

## Methodology

### Data collection, measures, and data analysis

The analysis of this research is based on primary data. The dataset is from a Work-from-Home study (Pfnür et al. 2021). Data collection was carried out by means of a written survey. The electronic questionnaire of the empirical study was distributed online with the software LamaPoll via the platform Clickworker. The empirical data basis is formed by the answers of the respondents. The data sample is of particular interest and potential as the results are based on industry-wide data in contrast to single-case studies, which are mostly used in existing literature in the field of NWW.

The survey addressed 2000 office and knowledge workers who perform at least part of their activities from home during the COVID-19 pandemic. From the aggregated dataset, which consisted of the responses generated from three survey waves (in June, August, and October 2020), only data from the second survey are analyzed in this paper. The survey was conducted from August 10th–14th, 2020. On the landing page of the survey there was a brief introduction on the goal of the study. The confidentiality and anonymity of the responses were emphasized. The mean survey duration was 33.5 min and 565 knowledge workers participated in this survey wave. These included participants from Germany, Austria, Switzerland, and the USA. Because the publication of the survey was through the Clickworker platform, calculation of a response rate is not possible. The Likert scales chosen in the survey provide metric data for the analysis.

Data cleaning took place in three steps using IBM SPSS Statistics (Sarstedt and Mooi 2011). First, all surveys that were answered within a shorter duration than seven minutes were excluded. The average response time is 32.9 min. In the second step, single outliers with a value above three standard deviations were removed. In addition, for the present study, only questionnaires were included in which the question about the current living country was answered as “Germany” (*n* = 429). After the data cleaning, missing values do not occur and missing data treatment and value treatment options can be ignored. Regarding the required sample size, a widely used rule of thumb for determining this is the “ten times rule of thumb” (Barclay et al. 1995; Hair et al. 2017). Another thorough assessment is provided by the statistical power tables documented in Hair et al. (2017). The given sample size exceeds both estimates of minimum sample size requirements and ensures a sufficient level of statistical power.

For this paper, PLS-SEM is chosen for the analysis. This method has received considerable attention in recent research (Ringle et al. 2018). In contrast to the more traditional CB-SEM, the focus of the paper and the model is on prediction and theory development, respectively (Richter et al. 2016), to understand increasing complexity by exploring theoretical extensions (Hair et al. 2019) of the ED–R theory. The statistical power of PLS is always greater than or equal to that of CB-SEM given a measurement model with sufficient quality (e.g., four indicators per construct) and more than 100 observations to achieve acceptable statistical power (Reinartz et al. 2009; Goodhue et al. 2012; Sarstedt et al. 2017), which also influences the decision. Considering the sample, the size is appropriate to choose this method as PLS-SEM works with small and large sample sizes (Hair et al. 2019). Furthermore, the path model is rather complex with its seven constructs. This paper focuses on the analysis of the target construct’s key sources of explanation and the relationship of resources and demands to productivity. In addition, research shows that PLS-SEM provides solutions when other methods do not converge or obtain valid results (Reinartz et al. 2009; Henseler et al. 2014; Sarstedt et al. 2016). The path modeling software SmartPLS 3 serves for the analysis (Ringle et al. 2015). The PLS-SEM algorithm settings are as follows: the weighting scheme is set to path and the abort/stop criterion is 10^−7 with 300 maximum iterations. The stop criterion changes present nine iterations before the PLS-SEM algorithm converges.

### Variable construction sample description

Items were combined from existing survey instruments and further developed. A detailed list of items with associated sources can be found in Appendix. A five–seven-point Likert scale was used for all items to measure perceived fit.

Table 1 reports the employee characteristics of the sample. The characteristics of the sample are representative for the respective society of Germany because the nonresponse bias analysis, consisting of a comparison of estimates from respondents to population values (Bose 2001), shows that the sample does not differ significantly from the target population in terms of its distribution across known variables such as gender or age.

**Table 1:** Respondents’ Demographic Characteristics. (Own illustration 2021)

Demographic Characteristic	Frequency (N = 429)	Percentage (%)
*Gender*		
**Male**	**262**	**61.1**
Female	166	38.7
Diverse Gender	1	0.2
*Age*		
18–20	13	3.0
**21–39**	**257**	**60.0**
40–55	131	30.5
56–68	28	6.5
*Relationship Status*		
Divorced	12	2.8
Married	142	33.1
**Relation**	**163**	**38.0**
Single	102	23.8
Widowed	1	0.2
N/A	9	2.1
*Level of Education*		
Hauptschule	7	1.6
Realschule	85	19.8
**Higher School Certificate (Abitur)**	**121**	**28.2**
Bachelor	82	19.1
Master craftsmen	6	1.4
Master	113	26.3
PhD	15	3.5
*Professional Status*		
**Employee**	**353**	**82.3**
Self-employed	47	11.0
Civil servant	15	3.4
Freelancer	14	3.3
*Position*		
Entrepreneur/Freelancer	46	10.7
Managing director	5	1.2
Management	65	15.2
Project manager	45	10.5
**Employee**	**244**	**56.9**
Temporary staff	4	0.9
Apprentice	10	2.3
Intern	2	0.5
Other	8	1.9
*Managerial Responsibility*		
Yes	92	21.4
**No**	**337**	**78.6**

Maximum values per demographic are printed in **bold**

## Construct validation and results

### Measurement models

The criteria evaluated in the following subchapter refer to reflective measurement models because the research model includes only this type of construct measurement. When using the bootstrapping procedure to derive *p*-values and BCa confidence intervals, and to examine the significance and relevance of coefficients, the criteria were for full bootstrapping using 10,000 subsamples (Streukens and Leroi-Werelds 2016).

For all indicators, the results show loadings above 0.708 (see Table 2), which is a desirable value for reflective specified construct indicator loadings. Therefore, the exceeding values indicate that the constructs explain more than 50% of the indicator’s variance (Sarstedt et al. 2017) and demonstrates a satisfactory degree of reliability (Chin 2010).

**Table 2:** Indicator Loadings, Mean Values, and Standard Deviations. (Own Illustration 2021)

	Outer Loading	Mean Value	Standard Deviation
*Isolation*			
Iso_1	0.915	2.275	1.068
Iso_2	0.909	2.357	1.114
Iso_3	0.858	2.765	1.148
*Family-Work Interference*			
FWI_1	0.950	3.573	1.378
FWI_2	0.854	3.508	1.402
FWI_3	0.926	3.909	1.645
*Equipment/Facilities*			
EF_1	0.801	4.550	1.738
EF_2	0.746	5.233	1.586
EF_3	0.826	4.946	1.435
*Building*			
Build_1	0.707	3.423	0.743
Build_2	0.914	3.231	0.639
Build_3	0.923	3.308	0.666
*Burnout*			
Burn_1	0.901	2.669	0.930
Burn_2	0.918	2.513	0.950
Burn_3	0.887	2.809	0.959
*Satisfaction*			
Satis_1	0.679	5.023	1.392
Satis_2	0.766	5.414	1.408
Satis_3	0.730	5.110	1.317
Satis_4	0.709	4.557	1.411
*Productivity*			
Prod_1	0.801	4.848	1.528
Prod_2	0.905	4.800	1.526
Prod_3	0.933	4.793	1.513
Prod_4	0.940	4.734	1.688

The extent to which items within an instrument measure different aspects of the same construct and yield the same answer each time they are administered (*ceteris paribus*), when all other things remain unchanged is tested with Cronbach’s α, composite reliability, and ρA. In general, higher values indicate higher reliability and vary between zero and one for all three measures (Hair et al. 2017). Results of the analysis (see Table 3) show for all three measures and all constructs values between 0.7 and 0.95, which is a recommended value range for satisfactory to good results. Overall, all items are identified as valid measures of the constructs.

**Table 3:** Internal Consistency, Reliability, and Convergent Validity. (Own Illustration 2021)

	Internal Consistency			Convergent Validity
	Cronbach’s α	ρ_A_	Composite Reliability	AVE
Isolation	0.875	0.879	0.923	0.800
Family-Work Interference	0.899	0.938	0.936	0.830
Equipment/Facilities	0.708	0.729	0.834	0.628
Building	0.814	0.881	0.888	0.813
Burnout	0.885	0.887	0.929	0.813
Satisfaction	0.705	0.732	0.813	0.521
Productivity	0.917	0.921	0.942	0.804

The convergent validity of each construct, “the extent to which a construct converges in its indicators by explaining the items’ variance” (Sarstedt et al. 2017, p. 16), also referred to as “communality,” is measured by the average variance extracted (AVE). For all constructs of the research model (see Table 3), the AVE metric for all items associated with their construct is above 0.50. Only the satisfaction value scarcely overreaches the recommended bound. This still indicates that the construct explains at least 50% of the variance of its items (Chin 1998; Hair et al. 2019).

The final step of the reflective measured constructs analysis assesses discriminant validity to analyze how strongly constructs differ empirically from one another. This includes how strongly a construct correlates with other constructs and how pronounced the indicators of a construct represent only this one construct. Following Henseler et al. (2015), the heterotrait–monotrait (HTMT) ratio of the correlations is assessed. The HTMT criterion is defined as “the mean value of the indicator correlations across constructs (i.e., the heterotrait–heteromethod correlations) relative to the (geometric) mean of the average correlations of indicators measuring the same construct” (Sarstedt et al. 2017, p. 17). The threshold value of the measurement is 0.9. The analysis results (see Table 4) show for all constructs HTMT ratios below 0.9 and can, therefore, be considered as unproblematic (Henseler et al. 2015). With the highest value of 0.833 between satisfaction and equipment/facilities, all values for the upper bound of the 95% bias-corrected and accelerated confidence interval are always lower than 0.850, which indicates significant results by even lower values than the more conservative threshold value.

**Table 4:** HTMT Ratios. (Own Illustration 2021)

	**Burnout**	**Equipment/Facilities**	**Family-Work Interference**	**Building**	**Isolation**	**Productivity**	**Satisfaction**
**Burnout**							
**Equipment/Facilities**	0.235 CI^95^ = 0.360						
**Family-Work Interference**	0.426 CI^95^ = 0.526	0.359 CI^95^ = 0.468					
**Building**	0.035 CI^95^ = 0.043	0.189 CI^95^ = 0.273	0.181 CI^95^ = 0.268				
**Isolation**	0.362 CI^95^ = 0.459	0.361 CI^95^ = 0.477	0.093 CI^95^ = 0.132	0.073 CI^95^ = 0.180			
**Productivity**	0.116 CI^95^ = 0.226	0.502 CI^95^ = 0.607	0.268 CI^95^ = 0.383	0.125 CI^95^ = 0.212	0.400 CI^95^ = 0.506		
**Satisfaction**	0.573 CI^95^ = 0.666	0.748 CI^95^ = 0.833	0.633 CI^95^ = 0.711	0.270 CI^95^ = 0.363	0.541 CI^95^ = 0.562	0.455 CI^95^ = 0.637	

*CI*^*95*^ presents the upper bound of the 95% bias-corrected and accelerated confidence interval

### Structural model

The quality of the measurement model evaluation results is satisfactory; hence, the PLS-SEM evaluation process continues with the structural model (Hair et al. 2013). To avoid undetected collinearity that could bias the regression results, latent variable scores of the predictor constructs in a partial regression are used to calculate the VIF values. This is important because structural model coefficients, also called “path coefficients,” for the relationships between the constructs, are derived from estimating regression equations (Hair et al. 2019; Sarstedt et al. 2017). The test of collinearity between the constructs (see Table 5) shows for the structural model exclusively values smaller than two. Thus, multi-collinearity is not an issue as VIF values should not exceed a value of five (Hair et al. 2011) or, even more conservatively, 3.33 (Diamantopoulos and Siguaw 2006) as this is an indicator of collinearity problems between predictor structures. Already with results in the value range from three to five such problems can occur (Mason and Perreault 1991; Becker et al. 2015), which is why in the present work a value of three is defined as an upper limit. Thus, from a prediction viewpoint, the regression results are not biased because no undetected collinearity was found between the structural model coefficients.

**Table 5:** VIF Values. (Own Illustration 2021)

	Burnout	Productivity	Satisfaction
Burnout		1.234	1.040
Family-Work Interference	1.003		
Isolation	1.003		
Equipment/Facilities			1.065
Building			1.027
Satisfaction		1.234	

The variance explained in each of the endogenous constructs is reviewed by analyzing R^2^, a coefficient of determination of the model’s in-sample explanatory and predictive power (Shmueli and Koppius 2011; Rigdon 2012; Dolce et al. 2017). Values range from zero to one. Depending on the study and discipline considered, accepted R^2^ values vary, but a rule of thumb for acceptance is 0.75 (substantial), 0.50 (moderate), and 0.25 (weak) as higher levels indicate greater explanatory power through better predictive accuracy (Henseler et al. 2009; Hair et al. 2011). Analysis results for this research show R^2^ values between 0.242 (burnout), and 0.467 (satisfaction) (see Table 6).

**Table 6:** R^2^ Values. (Own Illustration 2021)

	*R*^*2*^
Burnout	0.242
Satisfaction	0.467
Productivity	0.254

**Fig. 4:**
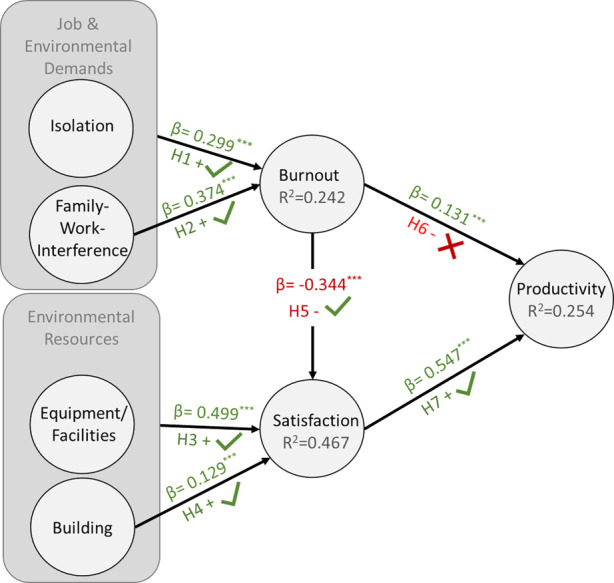
Research Model including Hypothesis and Structural Model Results. (Own Illustration 2021)

The statistical relevance and significance of the path coefficients are assessed with respect to the hypothesized relationships between the constructs (structural pathways). In terms of relevance, the path coefficients have standardized values approximately between minus one and plus one (Hair et al. 2019). The research model has seven path coefficients, five of which have a positive value and suggest a positive relationship (see Table 7). The path between satisfaction and productivity has the strongest relationship (0.547) because values that vary in strength and coefficients with a value closer to plus or minus one indicate stronger relationships. One path coefficient indicates a negative relationship between burnout and satisfaction (−0.344). For the confirmation of significance of a path coefficient, the number zero cannot be included between the limits of the confidence interval (5% and 95% probability of error level). The results show for all path coefficients significant coefficients on a 1% level. According to the path coefficients and their significance except for H6, all hypotheses can be confirmed (see Fig. 4). The values presented show that the model meets the quality criteria of the structural model and that the results can, therefore, be evaluated with valid content.

**Table 7:** Path Coefficients (Own Illustration 2021)

Hypothesis	Hypothesized Path	Path Coefficient	Confidence Intervals [2.5%, 97.5%]
*Burnout*			
H1	Isolation → Burnout	0.299***	[0.212; 0.384]
H2	Family-Work Interference → Burnout	0.374***	[0.275; 0.456]
*Satisfaction*			
H3	Equipment/Facilities → Satisfaction	0.499***	[0.428; 0.562]
H4	Building → Satisfaction	0.129***	[0.058; 0.199]
H5	Burnout → Satisfaction	−0.344***	[−0.416; −0.273]
*Productivity*			
H6	Burnout → Productivity	0.131***	[0.034; 0.229]
H7	Satisfaction → Productivity	0.547***	[0.457; 0.621]

***Significant at 0.01 level (2-sided), **significant at 0.05 level (2-sided), *significant at 0.1 level (2-sided)

## Discussion

### Theoretical contribution

The path coefficients of the structural model evaluation led to the conclusion that all four demands and resources of home office included in the model have a statistically significant influence on productivity when considering burnout and satisfaction as full mediating effects. Thus, except for H6, all hypotheses of the model are confirmed and the research question is answered. Equipment/facilities play a specifically crucial role in explaining satisfaction as the path coefficients has the strongest positive effect followed by the effects of family–work interference and isolation on burnout. The lowest effect has home on satisfaction. Burnout has a negative impact on satisfaction. Among the influences that burnout and satisfaction have on the target variable productivity, the relationship between satisfaction and productivity is higher than the surprisingly also positive impact of burnout on productivity.

This finding of equipment/facilities being a resource in home office with influence on satisfaction is consistent with previous studies in flexible workspaces that have found a positive correlation between access to needed technology and satisfaction (Van der Voordt 2004). Furthermore, it becomes clear that the trend in office building design regarding the design and functionality of modern furniture and high-tech ICT is also applicable to and helpful for the home office. Expanding further on the discussion, these results also provide an opportunity to extend the ED–R model. Roskams and Haynes (2021) provide a table with possible environmental demands and resources. Under the heading “aesthetic and ergonomic design,” their table lists “uncomfortable furniture” as an environmental demand. Based on the results, this paper extends the existing table by proposing the inclusion of equipment/facilities factors and building as environmental resources.

A surprising finding emerges in the relationship between burnout and productivity. The assumption that burnout has a negative influence on productivity in the home office cannot be confirmed. Because burnout is represented by exhaustion in this paper, the assumption mentioned in COR theory that excessive work demands in comparison to resources leads to burnout and a feeling of inefficiency cannot be supported (Hobfoll 2001). To the author’s knowledge, a positive relationship between burnout and productivity has not yet been demonstrated in research. In the present model with participants from the knowledge worker sector, it is suggested that the negative relationship may be due to absence from the office workplace and a lack of comparability. It is possible that although people suffer from exhaustion, they rate their productivity higher than they would if they could compare their performance with that of their colleagues. Another explanation lies in the time lag between measurement and effect. It is conceivable that working from home initially caused exhausted individuals to continue working, which eventually led to higher productivity. However, as the pandemic progresses and home office work expands, productivity might decline as exhaustion reaches a point where it is physically or mentally impossible for the worker to continue working productively. Lastly, the results of some studies show a higher risk for “boreout” when working in a home office (Pfnür et al. 2021). The effect of boreout, as a combination of boredom, lack of accomplishment, disinterest, and behavioral strategies aimed at appearing busy and hiding the fact that one is not working (Rothlin and Werder 2007), can be suspected as a possible substitute for the burnout effect.

Furthermore, Cropanzano and Wright (2001) argue that research on the correlation between satisfaction and productivity would be stronger if the operationalization of satisfaction included more than just job satisfaction. This claim was investigated in this paper, which resulted in high influence of satisfaction on productivity, thus supporting their proposition. Therefore, not only in the home office, but for workplace research in general, it is relevant to operationalize satisfaction more broadly than just with job satisfaction. In this way, a broader spectrum can be mapped to obtain a holistic picture. The scientific knowledge gained about the effects of the success of home office implementation on employee work behavior and company success paves the way for the necessary measures.

### Practical contribution

Several implications for companies and practitioners can be derived from the results of this work. These implications include developing recommendations for CREM in an integrative manner on how best to deal with the new situation during the COVID 19 pandemic in the short term and beyond with home offices. For a more detailed interpretation of the PLS-SEM results, an Importance Performance Map Analysis (IPMA) (see Appendix) completes the picture by providing more specific insights into the target variable (e.g., Höck et al. 2010; Hair et al. 2017). This method is utilized to identify the constructs that should receive highest priority for performance improvement (Ringle et al. 2015). Environmental resources and equipment/facilities as well as buildings have a positive influence on satisfaction. Improving these resources, therefore, leads to an increase in satisfaction and productivity among employees in the home office. Thus, the two resources form the most important starting point for CREM practitioners. As already recognized by Van der Voordt (2004), who mentions workplace comfort, ergonomics, and sufficient storage space as among the most important factors influencing productivity in flexible workplaces, it is important to pay special attention to and improve the aspects of equipment/furniture.

Real estate is often seen as a cost factor for companies. In the past, the top priority of CREM was, therefore, the optimal allocation of space. Instead of focusing operationally on cost reduction through space efficiency, the focus should be on increasing the benefits for the end users by investing in workplace development. Improved work results through increased productivity of employees illustrate the need for a change in thinking. This paper illuminates that space also has relevance for organizational outcomes in the new workspace at home as the two workplace characteristics, equipment/facilities and building, have a positive impact on employee productivity. CREM should take this finding from the home office lab as an opportunity to draw conclusions about the connection points of all workplaces and adapt them in the best possible way. With the confirmed influence of all four factors included in the model, this paper provides CREM with many starting points to increase employee productivity in the home office during COVID-19. Thus, the results of this paper should be considered by multiple sectors across corporate departments as they show that workplace characteristics influence productivity when working from home. The impact of the work environment, therefore, requires closer collaboration between the disciplines of HR and CREM.

For CRE managers, the following advice and recommended actions arise at three levels:

- First, work should be done to design and maintain employees’ **home offices** so that they provide physical, functional, and psychological comfort for employees.
- Mitigate environmental demands and enhance environmental resources by creating appropriate workspaces at home through a combination of top-down and bottom-up strategies (supporting Roskams et al.’s (2021) recommendation) through developing and actively implementing flexible work policies that allow employees to individually participate in the process of environmental design of the workspace.
- Involve the users of the workplace and use the strength of workers who individually know what equipment and furniture will help them create their work environment free from demands and rich in resources to increase satisfaction and productivity.
- Second, develop **strategies for multi-local working** as employees are reluctant to choose only one work location after having had the opportunity to experience the advantages and disadvantages of this work location during the home office period in the COVID-19 pandemic. The advantage of employees themselves knowing which work location is the best place to perform which activities can add up to the best possible work outcomes when a strategy of multi-locality is adopted.
- Third, face the pressure to **redesign office workplaces** forced by increasing flexibility and distribution of work. As a certain number of employees do their work in the home office and claim to be able to perform certain activities more productively there, the main task of offices will change in the future, as employees will only come there for certain other activities.
- Rethink traditional office spaces and orient the development of the work environment to changes in the way of working. Meetings might be held differently than in the past, with more virtual communication and collaboration; however, working from home is not expected to become a complete and effective substitute for face-to-face interaction (Nathan and Overman 2020). An office workplace for real physical or temporary interactions with mutual collaboration to create innovation will be needed. Gauger et al. (2022) identify communication and social interaction as two of the main significant predictors of work satisfaction in flexible workspaces. The creation of physical places for community and social interaction that do not exist at home should remain the goal to complement new flexible forms of work in the best possible way. In the future, the office needs to be a place of encounter. If it is possible to use the office workplace for joint professional communication, collaboration and social exchange, then the missing possibilities occurring in the home office can be compensated. The benefits are manifold, from meeting people with diverse skills and competences, building ties to creating knowledge spill-overs.

## Limitations and further research

The chosen research approach via PLS-SEM analysis in this paper investigates research questions with more weight. As with most surveys, the design of the questionnaire cannot completely rule out selection biases even though measures have been taken to reduce them as much as possible. The fluctuations of subjective perception to which the survey results are prone introduce a possible lack of measurement accuracy of the intended conditions or results and could lead to erroneous conclusions. The importance of researching home office outcomes for organizations is not diminished by the fact that data collection took place at a specific point in time. At the same time, recent issues, such as vaccination and overcoming the pandemic, are not yet reflected in the data, but need to be considered for long-term adjustments. Because the analysis took place during COVID-19, it may be helpful in future research to test the results for applicability outside of lockdown periods. To this end, a study building on this work could attempt to analyze the influence of social factors such as school closures and pandemic-related stress on the results. In addition, because the study is limited to the context of knowledge workers using a sample of German employees working from home, future research within different countries and comparisons between the results can be interesting to learn from best practices.

Furthermore, the study does not consider all possible environmental characteristics of home office work but rather concentrates on those identified as most relevant. For further NWW research on home office and its impact on organizational outcomes, the ED–R model should be used as a research base. Due to the high influence of equipment/facilities on employee satisfaction and, thus, on productivity, it is important for further research to consider these factors in greater detail. Therefore, future research should also include other factors of real estate, e.g., identify and specify important facilities. With increased use cases of the research model, the ED–R model can be targeted to have comparable importance in workspace research as the JD–R model has in stress research. This higher importance could lead to more comparable results and a clearer understanding of the advantages and disadvantages of NWW, such as home office. Based on an established theory, companies’ CREM departments can apply the findings to a greater extent.

## Conclusion

This paper provides important insights to better understand how home office factors influence the productivity of knowledge workers in Germany. It demonstrates the relevance of the four environmental characteristics (isolation, family–work conflict, equipment/facilities, building) for employees working from home. The paper provides a methodological approach for understanding and empirically measuring the influences on knowledge workers’ productivity through burnout and satisfaction during COVID-19. By building on the ED–R model, this paper contributes to establish a more inclusive and integrative framework for physical working environment research and offers new approaches to extend the model. Based on the conducted literature review and the PLS-SEM analysis of the research model, all hypotheses except for H6 are confirmed. When considering specific resources and demands that have an influence on organizational outcomes in the home office, the results show that equipment/facilities have the greatest influence. In addition, isolation and family–work interference have a positive influence on burnout. Equipment/facilities as well as the home have a positive influence on satisfaction. Both satisfaction and burnout influence employee productivity positively while burnout has a negative influence on satisfaction. Based on the results of this paper, companies and CREM can gain knowledge as to how to best focus on their employees’ workplaces to address the changes in the world of work, especially in the exceptional situation of the pandemic. The identified potential for improvement of the observed influencing factors should be used to maintain the satisfaction of home office employees and to control burnout as a decrease in productivity is a potential risk for companies and, consequently, their competitiveness. In addition, based on the results of this paper, CREM can implement appropriate approaches to provide a useful home office workspace during COVID-19 when available environmental resources exceed environmental demands. Looking at everyday life after the pandemic and to be prepared for future crises, the readiness of the CREM department to adopt location-flexible workplace options, such as the home office, opens up new ways to implement suitable solutions for the needs of knowledge workers. By combining theoretical and methodological elements from CREM and physical workspace research, this paper can provide an important step toward an influence-based approach to the home office.

## Figures and Tables

**Fig. 5:**
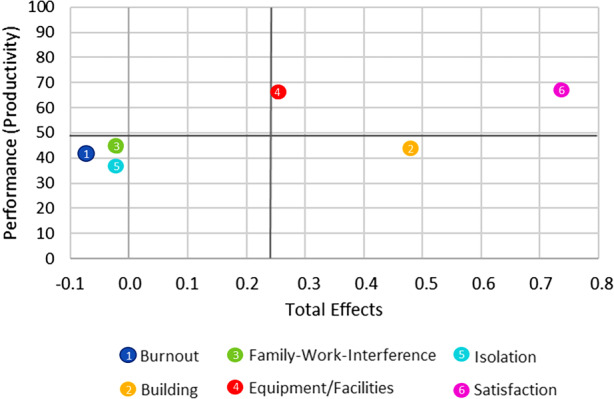
IPMA Map. (Own Illustration 2021)

## Appendix

### Operationalization

**Table 8:** Operationalization. (Own Illustration 2021)

Item	Constructs	Sources
Reflective		
*Isolation*		
Iso_1	I feel lonely at my workplace at home	(Bloom et al. 2015)
Iso_2	I feel isolated at my workplace at home	(Bloom et al. 2015)
Iso_3	At my workplace at home, I lack opportunities to socialize at and after work	(Bloom et al. 2015)
*Family-Work Interference*		
FWI_1	In most ways, my work-life balance is close to my ideal	(Diener et al. 1985)
FWI_2	So far, I have gotten the important things regarding my work–life balance	(Diener et al. 1985; Grawitch et al. 2013)
FWI_3	If I could live my life over, I would change almost nothing about my work-life balance	(Diener et al. 1985; Grawitch et al. 2013)
*Equipment/Facilities*		
EF_1	I have a full-fledged workplace in terms of furniture (including storage space)	(Maarleveld et al. 2009; BMFSFJ 2017)
EF_2	The technological equipment of your home office. I have full information and communication technology equipment (computers, printers, etc.)	(Møller-Jensen et al. 2008; Maarleveld et al. 2009; BMFSFJ 2017)
EF_3	The available rooms (equipment, furniture) support the work optimally	(Maarleveld et al. 2009; Gauger et al. 2022)
*Building*		
Build_1	The construction quality of the building in which I live	(Own research following Krupper (2013))
Build_2	Architecture of the building in which I live	(Own research following Krupper (2013))
Build_3	Interior of the building in which I live	(Own research following Krupper (2013))
*Burnout*		
Burn_1	I feel emotionally drained from my work	(Maslach and Jackson 1986; Moen et al. 2016)
Burn_2	I feel burned out by my work	(Maslach and Jackson 1986; Moen et al. 2016)
Burn_3	I feel drained at the end of the workday	(Maslach and Jackson 1986; Moen et al. 2016)
*Satisfaction*		
Satis_1	All in all, I am satisfied with my job	(Cammann et al. 1979, 1983; Bowling and Hammond 2008; Allen 2001)
Satis_2	I am satisfied with my home office	(Amérigo and Aragonés 1990; Gauger et al. 2022)
Satis_3	Your satisfaction with your life overall	(Diener et al. 1985; Bowling and Hammond 2008)
Satis_4	Your satisfaction with your financial situation	(Van Praag et al. 2003; Newman et al. 2008; Gray 2014)
*Productivity*		
Prod_1	Working in my home office makes it easier for me to do my work	(Own research following Krupper (2013))
Prod_2	Working in my home office increases my effectiveness at work	(Own research following Krupper (2013))
Prod_3	Working in my home office improves my productivity	(Own research following Krupper (2013))
Prod_4	I have the feeling that working at home is more productive than working at my professional office workstation	(Own research following Krupper (2013))

### IPMA productivity

**Table 9:** Importance and Performance Values. (Own Illustration 2021)

	Importance	Performance
Burnout	−0.093	41.480
Equipment/Facilities	0.308	65.405
Family–Work Interference	−0.022	43.686
Building	0.488	43.284
Isolation	−0.024	36.867
Satisfaction	0.760	68.808
Average Value	0.236	49.800
